# Supplementary material for: Assembly mechanism of a Tad secretion system secretin-pilotin complex
Source: Nat Commun. 2023 Sep 13;14:5643. doi: 10.1038/s41467-023-41200-1 (PMC10499894; doi:10.1038/s41467-023-41200-1)
Supplement: Supplementary file 1 — Supplementary Information [file 41467_2023_41200_MOESM1_ESM.pdf]

Structure of a bacterial Tad pilus secretion system secretin-pilotin complex

Matteo Tassinari[1], Marta Rudzite[2], Alain Filloux[2,3], Harry H Low[1]*

[1]Department of Infectious Disease, Imperial College, London, SW7 2AZ, UK

[2]Department of Life Sciences, Imperial College, London, SW7 2AZ, UK

[3]Current address: Singapore Centre for Environmental Life Sciences Engineering, Nanyang Technological University, Singapore

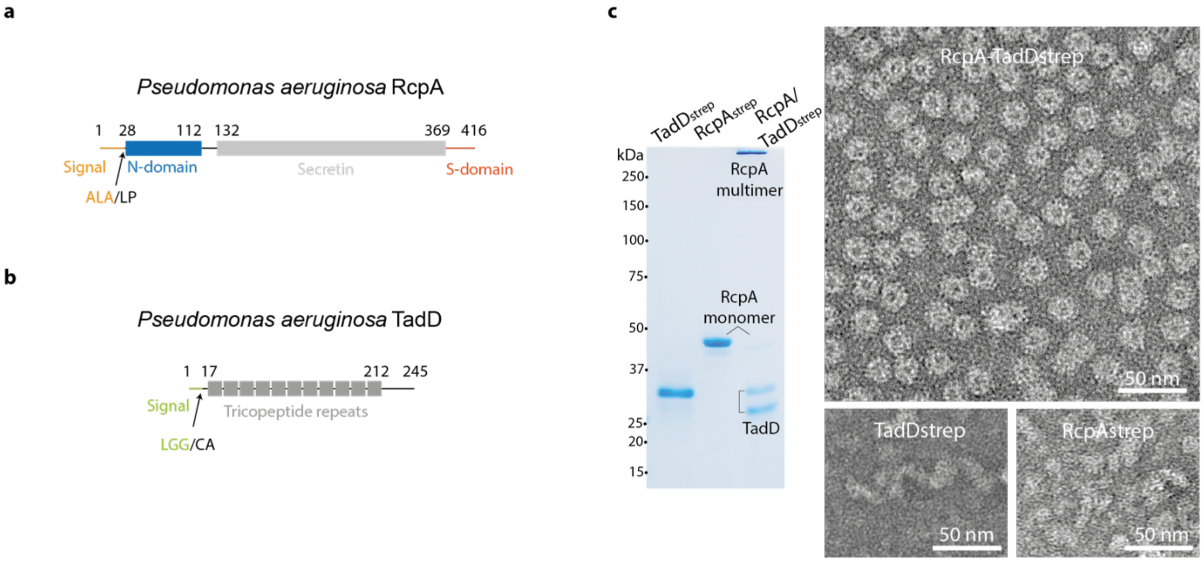

Supplementary Figure 1. Single and co-expression purifications of TadD with RcpA. **a**, Schematic showing domain architecture of *P. aeruginosa* RcpA. Note that RcpA in other systems often include a predicted N3 domain between the N-domain and secretin. **b**, Schematic showing domain architecture of *P. aeruginosa* TadD. It is predicted to be formed from 11 α-helices (grey squares) that fold as five tetratricopeptide repeats. **c**, SDS-PAGE analysis (left) and NS EM images (right) of RcpA and TadD purified separately and after co-expression. TadD purified alone forms helical filaments. The RcpA secretin (multimer) is resistant to SDS and thermal denaturation.

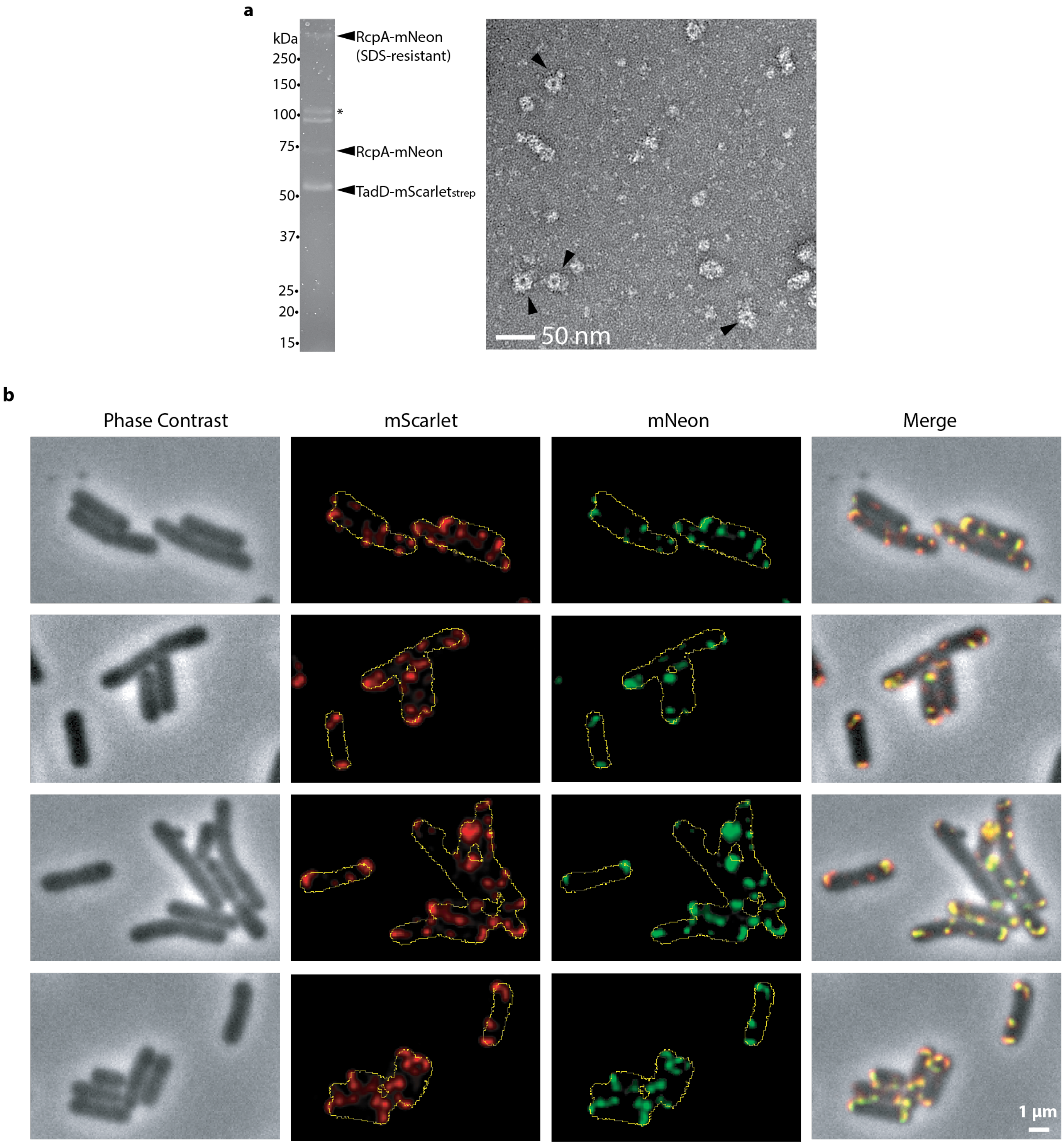

Supplementary Figure 2. RcpA and TadD co-localise when heterologously expressed in *E. coli*. a, Co-expression and purification of RcpA-mNeon and TadD-mScarlet$_{strep}$ form assembled secretins. (Left) SDS-PAGE illuminated at 470 nm showing Strep-Tactin column eluate peak fraction with corresponding NS electron micrograph (right). Asterisk indicates oxidised species of TadD-mScarlet$_{strep}$. **b**, Gallery showing additional typical examples of RcpA-mNeon and TadD-mScarlet$_{strep}$ co-localisation when heterologously expressed in *E.* coli, related to Figure 1.

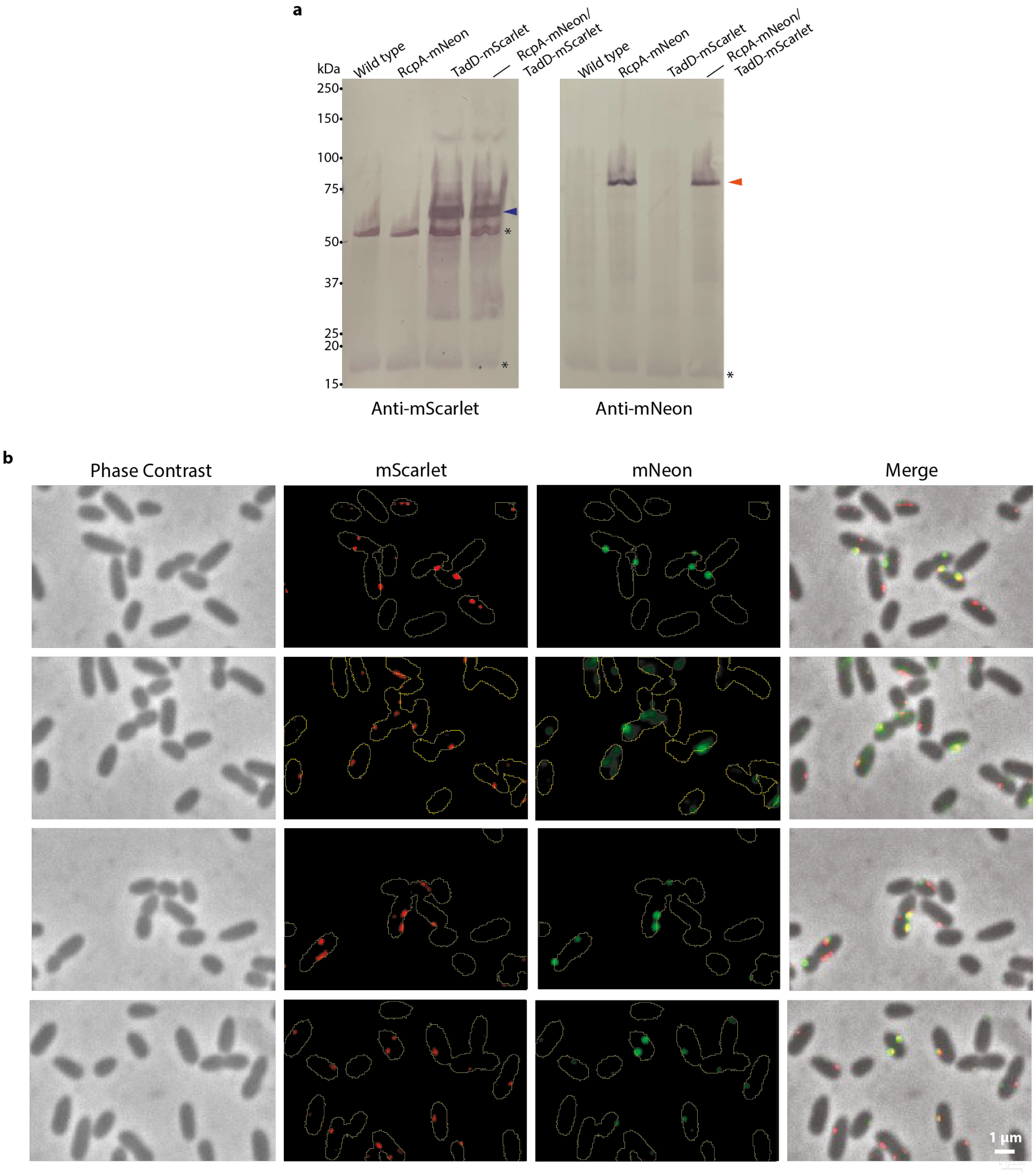

Supplementary Figure 3. RcpA and TadD co-localise in *P. aeruginosa*. a, Western blot against *P. aeruginosa* cell lysate strains using anti-mScarlet (left) or anti-mNeon (right) antibody. Asterisks indicate non-specific binding. Black arrow indicates expression of TadD-mScarlet at the expected height of 52 kDa. Red arrow indicates expression of RcpA-mNeon at the expected height of 68 kDa. For RcpA-mNeon/TadD-mScarlet we did not readily observe SDS-resistant oligomer consistent with secretin likely due to poor membrane transfer or increased sensitivity to SDS denaturation. **b**, Gallery showing additional typical examples of RcpA-mNeon and TadD-mScarlet co-localisation in *P. aeruginosa*, related to Figure 1.

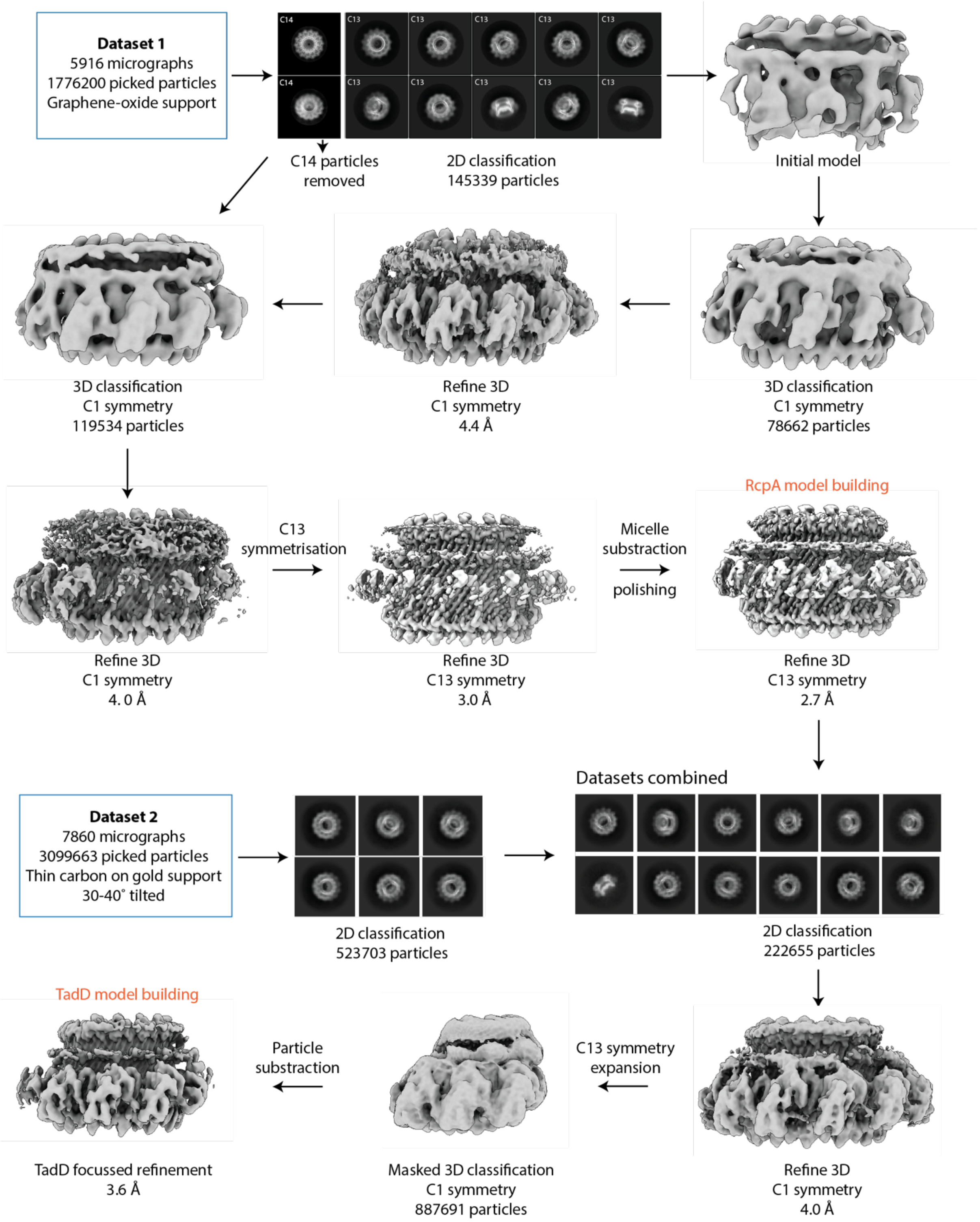

Supplementary Figure 4. $RcpA_{strep}$-$TadD_{FLAG}$ complex processing strategy for 3D reconstruction and refinement, related to Figure 2.

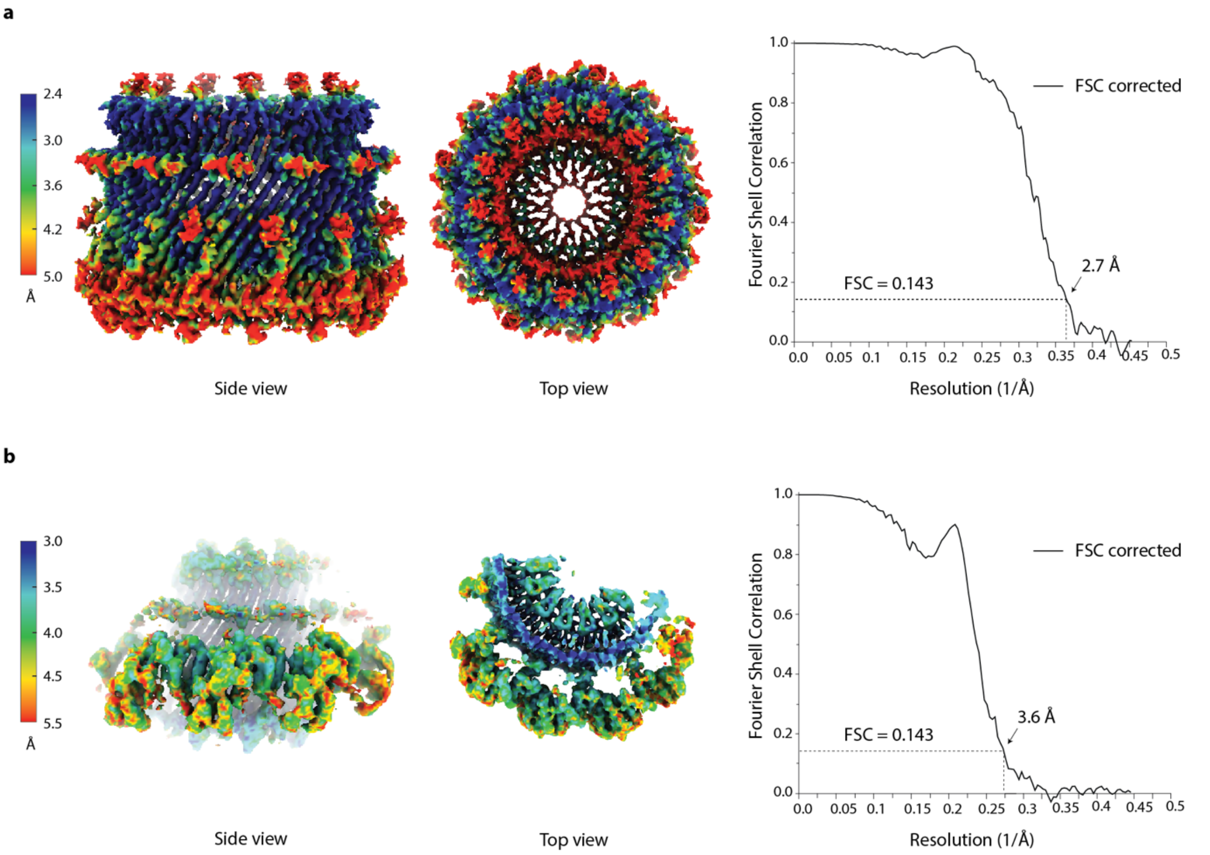

Supplementary Figure 5. RcpA$_{strep}$-TadD$_{FLAG}$ complex local resolution maps and FSC curves, related to Figure 2. a, Local resolution map for RcpA secretin contoured at 6σ and associated gold standard FSC curve. **b**, Local resolution map for the TadD focussed refinement map (TadD$_{FRmap}$) encompassing three RcpA subunits and three TadD subunits contoured at 6σ and associated gold standard FSC curve.

```
Pa    -MHRSTGI------------GVSRWLG-RLLGVALALPALA-------------------    27
Ye    --------------MKVARPRYLNQI--ALFFLCMI------------------------    20
Aa    ------------------MQNWNHTFGKKQLICCAV------------------------    18
Ap    ------------------MQ--INKFS-KTLLSSAL------------------------    15
Cc    -MSRPVNLPSVKASMSASRRLLTASVA-ALLALTSTAPVFADGPIGGSHTYRPPARVTRA    58
Vc    --------------------------M-KLLNKFSVLVVSL-------------------    14
Bp    MKQHKVGR------------HWAGWAM-ALACLGAAAPLAA-------------------    28

                              N0 domain
            H1                 S1     S2       S3       S4
Pa    ---LPQG------------CIELLAQAPRVDVVQGQQRDLRLAVPIQRLAIGDPKIADEL    72
Ye    ---------------ISFFIAFNRANAEPVYLSTGESYMIKTQEEIDTVFVSAAAIADYE    65
Aa    ---------------LGA-VFSLNAYAQNFSLDKGATQLVQTKEKIDTIFVSSPNIADYE    62
Ap    ---------------LGLLALSHSAMAKTFTLEQGQSQLIKTNAKIDTIFVSSSEVADYE    60
Cc    PAAAPAPVMVRAEDQVARIVMTADQTAATLELAKGKSAIVELPSEVRDLLVTNPQIADAV    118
Vc    ---LP--------------CY-AFAE-SPLVLHVNQNQIITVDGDIQDVFVSDPKVVVV-    54
Bp    ---QPAA------------PA-GAAQARELLLEVKGQQPLRLDAAPSRVAIADPQVADVK    72
                                   . :      :        : :    :.

                              N0 domain
                 S4        S5          S6
Pa    LVD-G----RGFLVSGKEQGSTSLLISTGCSPEPMRSLV---------------------    106
Ye    LVG-----KNSIIVYAKQEGTAEFILFNQNHHPIKKSAILVDNTI---TAAHKRIRLEYP    117
Aa    ILD-----DNTFIIYAKEEGRTEVTAFGADGRPLTSDTVNVDSVVTSIADTNKQLKSRFP    117
Ap    ILD-----DNSFMLYAKAEGRSEVIAFDANGTPLTEDFVNVNNAINNIAATNQQIQTRFP    115
Cc    LRD-----KRRIYIVGLAEGTTDAAFFDTAGRRILSLSIRVSQPVDQLAA---MLGKILP    170
Vc    ----HAPSARHVMVSGKEIGSTDLLILGDNAQTLAHYKLNVNADLSELER---AVQHAFP    107
Bp    VLAPGVGRPGEVLLIGRQAGTTELRVWSRGSRDPQVWTVRVLPQV----------QAALA    122
                 . : .   * :.               :

Pa    ------EVEGRGSVDT-------------RGAPAFTVG----------------------    125
Ye    ESDIEINKIGDSYILTGTVETEEAKETIASIIG-EAIGSEKTI----------ASNSKKD    166
Aa    NTNLSVKKVGKAYVIEGKARSQEESDEVRRIVG-EALGSGRKV----------TETKL--    164
Ap    NSNLTVKKVGKAYVLEGKAKNAAESDEINRIVG-ESLGAGKKV----------IETKLKH    164
Cc    DAKITVSPIRDSVVLSGTVR------------------------------------TASEAE    196
Vc    EAKIRFAYSKGAIVVMGEVANPLQAHEVLSMASGFARSLQKPEQQTLNMPANPASNGGRD    167
Bp    R----RGVGGGAQVDM-------------AGDSGVVTGM---------APSAEAHRGAAE    156
                 . :

                                         Secretin core domain
                                                S7
Pa    -------------AAE--------ELPNQVQTDIRFVEVSRSKLKQASTSFVRRGGNL--    162
Ye    SDG--NSQYFNSPEYSRVVNKIKLPGSNQVNVKLTIAEVSKDFSENIGMDWSTIGNF---    221
Aa    --GEDSLPFLDKYHYDGVVDNANIADTTQINVKLSVVEVNKKLSEAMGINWSHVAGSGPL    222
Ap    NDGEENVPFLDKYQYEGVINNANEENATQINVKLTVAEVNKTFSDEIGINWSNLSGNFFR    224
Cc    SAA--RIAAQFVGSPEKVLNMISVAGKDQVMLQVRIVEVQRNVIKQLGVDLNAVIGQLGE    254
Vc    SGA--AKPGGGIGDYEFVVNQLKTQGSAQVNISIRIVEMERATSEQLGLRWSSIGNMR--    223
Bp    AAA--A----AAGGNDKVVDMSQINTSGVVQVEVKVVELARSVMKDVGINFRADS-GP--    207
                     .             :  .: ..*: :   .  .

                          Secretin core domain
         S8
Pa    --WVL------GAPGSL-G----DIK------------VNADGSGLGGTFGT--------    189
Ye    -----SGSFQFFKP--------------------------------NG------------    232
Aa    VGGNFGFGGGFNGN--------------------------------QG------------    238
Ap    NLGNAAIQGNFNKN--------------------------------GG------------    240
Cc    TQYTFGMAPGYGVNGSLLGGVTGGYKMDTTKQPVMQTPCTGPGWTAGSLCPVVARGGGNS    314
Vc    --WGT------WDT--L-Q--------------------TALGATPGNKFPT--------    244
Bp    --WSG------GVS-LL-P------------------------DLASGGMFGM--------    226
                                                 .

                          Secretin core domain
          S9            S10              S11        S12
Pa    GSSGFNLIFGGG---KWLSFMNALEGSGFAYTLARPSLVAMSGQSASFLAGGEFPIPVPN    246
Ye    ----LN-K---FNAKGISALVHAINDDSIARVLAEPNLSVLSGESASFLVGGELPLVNTT    284
Aa    ---VLR-L----DAKGISAFINALDNQSNGKVLAEPNISMLSGETADILVGGEIPFAQRD    290
Ap    ---QLA-L---VNSNNLNVLISALDNQNNGKILAEPNISMLSGETADILVGGEVPFVQRD    293
Cc    DTATIQSTAGDPGLNSAKGMIQAFERVGLVRTLAEPNLAAVSGESGKFLVGGEFPVPTGS    374
Vc    NT--LPSGNGQH---GLNVIIDALVENSLVNVLAEPNLTAKSGEAATFMSGGEFPFPVDN    299
Bp    ------LSYTSR---DFSASLALLQNNGMARVLAEPTLLAMSGQSASFLAGGEIPIPVSA    277
                          :  :   .    **.*.:   **:. :: ***.*.
```

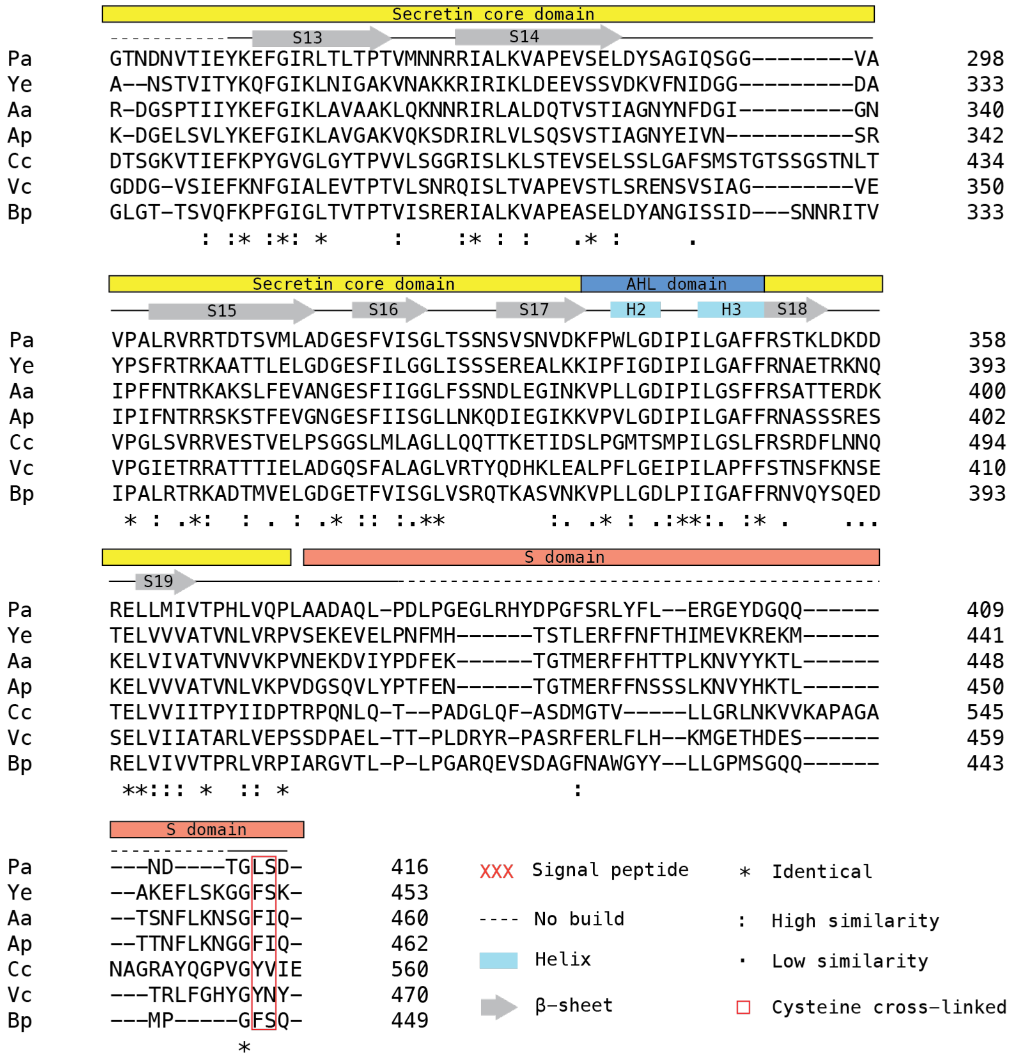

Supplementary Figure 6. RcpA secondary structure assignment and sequence alignment with RcpA from other TadSSs. Sequences were aligned using Clustal Omega and include those from *Pseudomonas aeruginosa* (Uniprot code Q9HW96), *Yersinia enterocolitica* (Uniprot code A0A8B6KXR4), *Aggregatibacter actinomycetem*comitans (Uniprot code E1CK52), *Actinobacillus pleuropleumoniae* (Uniprot code H6T5D2), *Caulobacter crescentus* (Uniprot code Q9L717), *Vibrio cholerae* (Uniprot code Q87I85), *Bordetella pertussis* (Uniprot code 0A0T7CLD4).

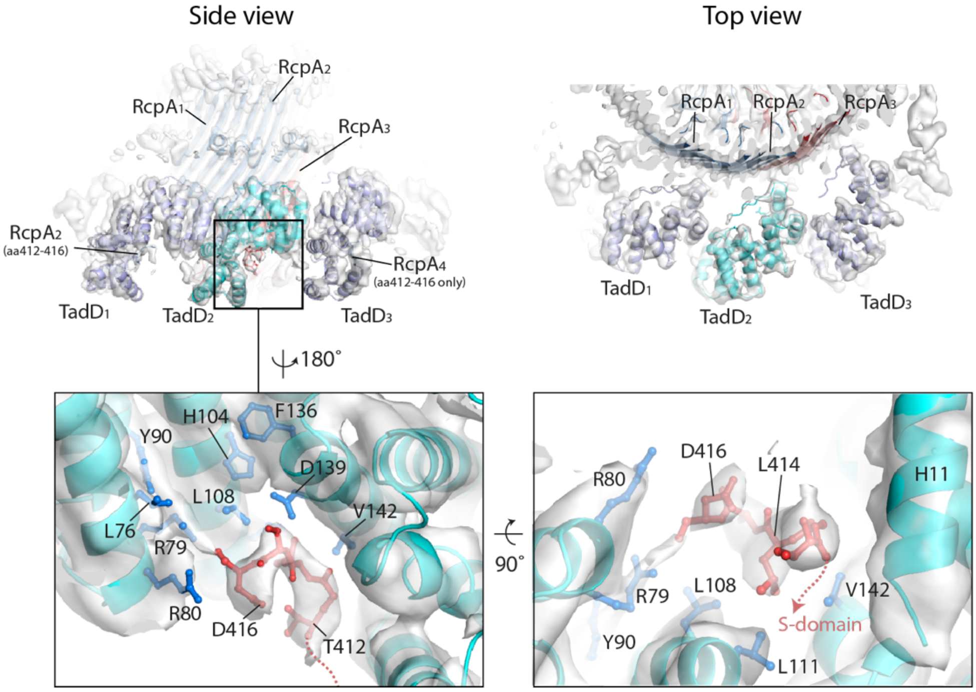

Supplementary Figure 7. TadD model build within TadD$_{FRmap}$. The map for TadD model building was obtained with a symmetry expansion and focussed refinement strategy (termed TadD$_{FRmap}$). Three TadD and four RcpA chains were needed for a complete build of three asymmetric units. Note that for one of the RcpA chains (RcpA$_4$) only amino acids 412-416 were required to complete the build of the TadD$_3$ subunit. Zoom boxes show build for the RcpA S-domain (RcpA$_{aa412\text{-}416}$) within the TadD groove with selected side chain detail shown for clarity.

```
Pa      ---MKALIG--------IGLCAALLGGCA--ALPGRD---------GPRECSQQ------      32
Ye      --MSKKISIT-----ITTLLFTVVLSGCSTH---NSI---------SSKEFYY-------      34
Aa      --MYSKSLKN-----VLLCGMILSVTACSAVLNKKPL---------SADKLAA-------      37
Ap      --MSSKLLKT-----IFVSTVLLSLSACSNLTA--NL---------SPAQKAE-------      35
Bp      MTTKPRLCTALLTLAIAVSASAPVLAQSMAGGSAKNRYANQTKLDPYPPEGGWQG-----      55
Vc      --MSPKFTAI-LPKPLWLLLCASLLVGCS--GTANKAAQHTESNARALAEASFNYGQYAQ      55
Cc      MCRKRALIAT-VLAPIALVLATPVFAA----DPPKADAGKTAKAAPAPVKASAQQ----R      51
                                .                        :

                                    H1                H2
Pa      ---------------------LGQEQELQ-------MNMVRDMIREGRLHAALANLESMP      64
Ye      RESILLKA-----NNYPGLITLYRDKLKSKEDDSVRLKLANAYYLTGDIKSSLYYLRPIS      89
Aa      KEALYQST-----NNNDALITMYRDGLKNKEDPLTRYKLSEIYYKKGDSSSSLLYLQPLL      92
Ap      QEKLYEDT-----KNYSALISLYRNQLKLNQDPLVQYKLAKSYYLSGDSKSSLLYLEPLR      90
Bp      -----L-------ANLLEAAKPGVDTRLDPTPSQITDR-IEELLNRGANEEALSLIERRE      102
Vc      AESQYLKLLASAPNNLHYQLMLGRSQYHQ-----------------DKKESAMAKLRHVT      98
Cc      TEARRLDPL-------AQAAFWGAEFEVDPADAEAGAGLAKALRALGRADEA---AIAAT      101
                                .                     .    :

                   H3                  H4                  H5
Pa      PGLLD-VREERALILR--RIG---DP--RARAEYQAL----LETCKAPEAHHGLGLLALR      112
Ye      HKEN----ASIYMLQT--KNLISNNDNATAKIVVNKL---LAISPDNAEAHNLNGI---I      137
Aa      TNSGQ-LSERAKILQA--RNLNQLKRYQEALEVENSL---LTTSPKNGEVYNLRGV---T      143
Ap      FKQLP-FNEDIELLYI--RNQIQAGAYNEAYLAATDL---IFQSPRSSEAYNLRAI---S      141
Bp      AAIKGQRGADVQLMFQHARALAALGRADEAIAVYSDM---TTQFPELPEPWNNLAALYAS      159
Vc      LAEDP-IAADAAVYLG--RYLLEAGRTAESVAVYQLGLPKAQQNSVKAQLHNGLGVALLE      155
Cc      KGLIA-HPDDTGLLLE--LAR---AHIARGQGFYAIE-PARKVAALSPKDWRPLTLLGVA      154
                    :                .                  :  .

                    H6                H7             H8
Pa      NGD---SARAVLELREAARLRPTESRFRNDLGVALLKRGDRVGARFEFITALELQQGGKL      169
Ye      LANDGEINKAEAAIEKSRSLFISDEIAMNNLAVTAMLDDRYSDAVRILLPDYLAGKRGSL      197
Aa      YALMGNPNKANEDINKAREYFLNDAVAVNNMAMLSIINGDYRNAVSLLLPQYLNGVREQR      203
Ap      LAQLGKLAAAEKDLNKSRELFIHDTIAINNLAMLSILNNDYRNAANLLLPQYLNGSKDSR      201
Bp      R---GELERAQDSLQMALRANPDYAAARANLGDIQLMM-----------ALRTYR-----      200
Vc      ----TNLEKARQELQQAITLAPDSPYFRSNLALSYLHDGQIKTAREVFTPLLAYRELPTQ      211
Cc      YEQAERDEEAEAAHRQAIALAPNEATPMANHAMHLAARGDLAGAEAELRRAVTLPSAGIQ      214
                 *    . :            : .

             H9          H10                                H11
Pa      PATNLLGLLYLQGDREDAQRLIER-------------LQ------L---DARDIRAAEAR      207
Ye      MLHNLVFSLVQLGDTQYAKKIIVAEKMAENPDQLILALSQVTNLRQERLPER*-------      249
Aa      LIHNLVFALVKNNEIDYAKDIIVKENINTSPDDLVNALKKTERVSNNIAR*---------      253
Ap      LIHNLVFALVKAGDTEYALDIIRKEHLNSSPEDLVNALQKTEKSPK-VGR*---------      250
Bp      -QEEARGVPGMKARVEEIESLLKD-------------KQPK*------------------      227
Vc      VELNFALLLLAENQQDQARALLSR-------------HMPTSEVERDLATLRARLNAIQV      258
Cc      VRQNLALVVGLQGRLPEAEKLVRA-------------DLPPEQVANNLAYLRAAVGQAGQ      261
           :                ::

Pa      ARSWGAVPTPGAAPASDDPLAELPAEANMHTAMANEAP*      245
Ye      ---------------------------------------      249
Aa      ---------------------------------------      253
Ap      ---------------------------------------      250
Bp      ---------------------------------------      227
Vc      AQ*------------------------------------      260
Cc      SRSWDAMRAGGGR*-------------------------      274
```

XXX Signal peptide   * Identical   □ Cysteine cross-linked

---- No build   : High similarity

Helix   . Low similarity

Supplementary Figure 8. TadD secondary structure assignment and sequence alignment with TadD from other TadSSs. Sequences were aligned using Clustal Omega and include those from *Pseudomonas aeruginosa* (Uniprot code Q9HWA1), *Yersinia enterocolitica* (Uniprot code A0A8B6KWP1), *Aggregatibacter actinomycet*emcomitans (Uniprot code E1CK58), *Actinobacillus pleuropleumoniae* (Uniprot code H6S0H2), *Caulobacter crescentus* (Uniprot code A0A0H3CAK5), *Vibrio cholerae* (Uniprot code A0A366A0T3), and *Bordetella pertussis* (Uniprot code A0A0E8DJC0).

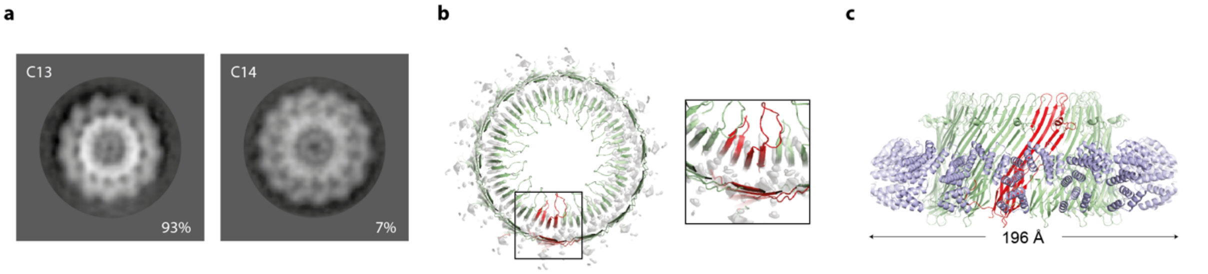

Supplementary Figure 9. *P. aeruginosa* RcpA-TadD complex with C14 symmetry. a, NS EM class averages of *P. aeruginosa* $_{strep}$RcpA-TadD complex with C13 and C14 symmetries obtained from *E. coli* heterologous expression and purification. Note here RcpA has an N-terminal strep tag used for purification to verify that the C-terminal strep tag used elsewhere does not influence secretin stoichiometry. **b**, Generation of RcpA$_{strep}$-TadD$_{FLAG}$ complex C14 symmetry model. RcpA$_{strep}$-TadD$_{FLAG}$ complex end views (0-20˚) were reconstructed to generate an anisotropic map into which fourteen copies of the RcpA-TadD asymmetric unit, as derived from the C13 symmetry structure, were fitted. (Right) Zoom box relates to boxed area outlined in left panel. **c**, Side view of the RcpA-TadD complex with C14 symmetry.

Table S1. Cryo-EM data collection, refinement and validation statistics

Data collection	Dataset 1	Dataset 2 (tilted)	Model RcpA-TadD C13
Electron microscope	Titan Krios	Titan Krios	
Voltage (kV)	300	300	
Pixel size (Å)	1.1	1.1	
Electron exposure (e−/Å2)	50	50	
Defocus range (µm)	0.8-2.5	1.0-2.5	
Images	5,892	7,323 – 30˚ tilt; 537 – 40˚ tilt	
**3D reconstruction**			
Final particles	119,534	429,217 - Carbon, 30˚ tilt<br>51,126 - Carbon, 40˚ tilt<br>407,348 - Graphene oxide	
Resolution (Å)	2.7	3.6	
FSC threshold	0.143	0.143	
B factor (Å2)	-68.5	-100	
**Refinement**			
Model	RcpA aa131-383	TadD aa29-212/RcpA aa412-416	
Chains	13	7	26
**Model Composition**			
Total atoms	46527 (Hydrogens: 23322)	19629 (Hydrogens: 9801)	85059 (Hydrogens: 42471)
Total residues	3107	1287	5577
**R.m.s deviations**			
Bond length (Å)	0.002	0.003	0.003
Angles (°)	0.479	0.672	0.624
**Validation**			
MolProbity score	1.05	1.74	1.54
Clashscore	2.39	9.37	8.62
Poor rotamers (%)	0.00	0.68	0.58
**Ramachandran plot (%)**			
Outliers	0	0	0
Allowed	2.13	3.64	2.38
Favoured	97.87	96.36	97.62
**B-factors/ADP**			
Minimum	13.92	13.92	13.92
Maximum	137.02	187.90	187.90
Mean	61.49	93.84	93.84
**Model vs. Data**			
CC (mask)	0.77	0.70	0.69
CC (volume)	0.76	0.67	0.67

Table S2. Strains used and generated

Strain	Plasmid	Purpose
E. coli C43 pspA:kan^r (DE3)	*pASK_rcpA.strep, pCDF_tadD.FLAG*	Cryo-EM
E. coli C43 pspA:kan^r (DE3)	*pASK_strep.rcpA ,pCDF_tadD*	Biochemistry, NS-EM
E. coli C43 pspA:kan^r (DE3)	*pASK_strep.rcpA_aa1-412, pCDF_tadD.FLAG*	Biochemistry, NS-EM
E. coli C43 pspA:kan^r (DE3)	*pASK_strep.rcpA_aa1-399, pCDF_tadD.FLAG*	Biochemistry, NS-EM
E. coli C43 pspA:kan^r (DE3)	*pASK_rcpA.strep*	Biochemistry, NS-EM
E. coli C43 pspA:kan^r (DE3)	*pCDF_tadD.strep*	Biochemistry, NS-EM
E. coli C43 pspA:kan^r (DE3)	*pASK_rcpA, pCDF_tadD.strep*	Biochemistry, NS-EM
E. coli C43 pspA:kan^r (DE3)	*pASK_rcpA L414C, pCDF_tadD.strep*	Biochemistry, NS-EM
E. coli C43 pspA:kan^r (DE3)	*pASK_rcpA L415C, pCDF_tadD.strep*	Biochemistry, NS-EM
E. coli C43 pspA:kan^r (DE3)	*pASK_rcpA, pCDF_tadD D139C.strep*	Biochemistry, NS-EM
E. coli C43 pspA:kan^r (DE3)	*pASK_rcpA L414C, pCDF_tadD D139C.strep*	Biochemistry, NS-EM
E. coli C43 pspA:kan^r (DE3)	*pASK_rcpA L415C, pCDF_tadD D139C.strep*	Biochemistry, NS-EM
E. coli AI	*pCDF_tadD.mScarlet.strep_rcpA.mNeon*	Fluorescence, NS-EM
E. coli AI	*pCDF_tadD.mScarlet.strep*	Fluorescence
E. coli AI	*pCDF_rcpA.mNeon*	Fluorescence
E. coli AI	*pCDF_tadD.mScarlet.strep_ rcpA_aa1-412 .mNeon*	Fluorescence
P. aeruginosa PAO1 rcpa:*rcpA.mNeon*	*n.a.*	Western blot
P. aeruginosa PAO1 *tadD:tadD.mScarlet*	*n.a.*	Western blot
P. aeruginosa PAO1 *rcpA:rcpA.mNeon tadD:tadD.mScarlet*	*n.a.*	Fluorescence, Western blot
P. aeruginosa PAO1 *tadD:tadD.strep*	*n.a.*	Western blot, NS-EM
P. aeruginosa PAO1	n.a.	Western blot
E. coli 1047 pRK2013	n.a.	DNA conjugations
E. coli CC118λpir	n.a.	DNA conjugations
E. coli Top10	n.a.	Cloning

Table S3. Primers

Construct	Primers	Template
pASK_rcpA.strep	FW_CCGCAGTTCGAAAAATAATAAGCTTTGTCGGATTAAGCTTGACCTGTGAA<br>RV_TGCTCCGATGCATTTTTTGCCCTCGTTATCTAGATTTTTGTCGAAC	pASK backbone
	FW_ATGCATCGGAGCACCGGGATAGGCGTGTCGCG<br>RV_TTTTTCGAACTGCGGGTGGCTCCAAGCGCTATCCGACAGACCGGTGTCGT	*P. aeruginosa* PAO1 gDNA
pASK_rcpA	FW_ACCGGTCTGTCGGATTAAGCTTTGTCGGATTAAGCTTGACCTGTGAAGTG<br>RV_TTAATCCGACAGACCGGTGTCGTTCTGCTGGCCGTCGTACTCGCCGC	pASK_rcpA.strep
pASK_strep.rcpA	FW_ATGGAGCCACCCGCAGTTCGAAAAGGGCTGCATCGAGTTGCTGGCGC<br>RV_CGAACTGCGGGTGGCTCCATGCGCTCTGCGGCAAAGCCAGTGCCGGC	pASK_rcpA
pASK_strep.rcpA aa1-412	FW_AACGACACCTAAGGTCTGTCGGATAGCGCTTGGAG<br>RV_CGACAGACCTTAGGTGTCGTTCTGCTGGCCGTCGTAC	pASK_strep.rcpA
pASK_strep.rcpA aa1-399	FW_CGCCTGTACTAATTCCTCGAGCGCGGCGAGTACGACG<br>RV_CGAGGAATTGTACAGGCGCGAGAACCCCGGATCG	pASK_strep.rcpA
pASK_rcpA L414C	FW_ACACCGGTTGCTCGGATTAAGCTTTGTCGGATTAAGC<br>RV_TCCGAGCAACCGGTGTCGTTCTGCTGGC	pASK_rcpA
pASK_rcpA L415C	FW_CCGGTCTGTGCGATTAAGCTTTGTCGGATTAAGCTTGACC<br>RV_TAATCGCACAGACCGGTGTCGTTCTGCTGG	pASK_rcpA
pCDF_tadD.FLAG	FW_GACGATGACGACAAGTAATGCTTAAGTCGAACAGAAAGTAATCGTATTGT<br>RV_CGATCAGTGCTTTCATGGTATATCTCCTTATTAAAGTTAAACAAAATTAT	pCDF
	FW_ATGAAAGCACTGATCGGCATCGGCCTGTG<br>RV_CTTGTCGTCATCGTCTTTGTAGTCGGATCCGGGCGCCTCGTTGGCCATCG	*P. aeruginosa* PAO1 gDNA
pCDF_tadD.strep	FW_CCACCCGCAGTTCGAAAAATAATGCTTAAGTCGAACAGAAAGTAATCG<br>RV_TCGAACTGCGGGTGGCTCCAGGATCCGGGCGCCTCGTTGG	pCDF_tadD.FLAG
pCDF_tadD D139C.strep	FW_TCCGCAACTGCCTGGGGGTCGCCCTGCTCAAG<br>RV_CCCAGGCAGTTGCGGAAGCGGCTCTCGGTC	pCDF_tadD.strep
pCDF_tadD.mScarlet.strep	FW_CAGCTGCATGGATCCTGGAGCCACCCGCAGTTCG<br>RV_TCCGCCACCTGCCGCGGCGGGCGCCTCGTTGGCCATCGC	pCDF_tadD.strep
	FW_GCCGCGGCAGGTGGCGGAGTGTCCAAGGGCGAAGCAGTGATCAAAG<br>RV_CCAGGATCCATGCAGCTGGCACGACAGGTTTCCC	17ACJD5P-mScarlet_pMA[illegible]T
pCDF_tadD.mScarlet.strep_rcpA.mNeon	FW_TACAAGTAATAATTAACCTAGGCTGCTGCCACCGCTGAG<br>RV_GGCAGCTGCCCCGCCTCCATCCGACAGACCGGTGTCGTTCTG	pCDF_tadD.mScarlet.stre[illegible]_rcpA
	FW_GGAGGCGGGGCAGCTGCCGTGTCCAAGGGCGAAGAGGATAACATGGC<br>RV_GGTTAATTATTACTTGTACAGTTCATCCATGCCCATCACATCGGTG	17ACJD6P_mNeonGreen[illegible]MA-T

Construct	Primer	Template
pCDF_tadD.mScarlet.strep_ rcpA aa1-412.mNeon	FW_CGACACCGGAGGCGGGGCAGCTGCC<br>RV_CGCCTCCGGTGTCGTTCTGCTGGCCGTC	pCDF_tadD.mScarlet.strep_rcpA.mNeon
pCDF_rcpA.mNeon	FW_TACAAGTAATAATTAACCTAGGCTGCTGCCACCGCTGAG<br>RV_GGCAGCTGCCCCGCCTCCATCCGACAGACCGGTGTCGTTCTG	pCDF_rcpA
	FW_GGAGGCGGGGCAGCTGCCGTGTCCAAGGGCGAAGAGGATAACATGGC<br>RV_GGTTAATTATTACTTGTACAGTTCATCCATGCCCATCACATCGGTG	17ACJD6P_mNeonGreen_pMA-T
pKNG101_tadD.mScarlett	FW_CGTCGAACCTGGGGATCCGTCGACCTGCAGG<br>RV_GCACCATCATTCTAGAGTCTTTGTTTTGACGCCATTAGCGTACGTAACAATCC	pKNG101_rcpA.sfGFP
	FW_AGACTCTAGAATGATGGTGCTGCTGTTCCCGGC<br>RV_GATCCCCAGGTTCGACGCGCTGGCTATGAAACTGCG	pASK_tadOperon_tadD.mScarlett
pKNG101_rcpA.mNeon	FW_ACTGTACAAGTAAGAGGCGCGCATGAACCAGAACTTCG<br>RV_CTTGGACACTCCTCCTCCTGCTGCTGCATCCG	pKNG101_rcpA.sfGFP
	FW_AGGAGGAGGAGTGTCCAAGGGCGAAGAGGATAAC<br>RV_GCGCCTCTTACTTGTACAGTTCATCCATGCCCATCACATCG	17ACJD6P_mNeonGreen_pMA-T
pKNG101_tadD.strep	FW_ATGGAGCCACCCGCAGTTCGAAAAGTGAGCCGGCGCCGGAGAGGT<br>RV_CGAACTGCGGGTGGCTCCATGCGCTGGGCGCCTCGTTGGC	pKNG101_tadD.mScarlett

Table S4. RcpA-mNeon and TadD-mScarlet co-localisation in *P. aeruginosa*

**Image 1 (Figure S3)**	**Cells number**	**colocalizing mScarlet centers**	**colocalizing mNeon centers**	**Pearson`s coefficient**
ROI 1	15	8/22	8/14	0.658
ROI 2	24	6/16	6/12	0.711
ROI 3	13	5/13	5/12	0.701
ROI 4	23	7/18	7/18	0.647
**Image 2**				
ROI 1	12	5/9	5/7	0.93
ROI 2	12	6/13	6/9	0.712
ROI 3	10	4/10	4/6	0.712
ROI 4	8	3/13	3/6	0.78
**Image 3**				
ROI 1	11	7/20	7/15	0.727
ROI 2	15	6/19	6/19	0.838
ROI 3	11	8/21	8/10	0.814
ROI 4	11	3/11	3/8	0.771
**TOTAL**	**165**	**68/185**	**68/136**	**Average 0.783**
**Foci/cell**		**1.12**	**0.82**	
**Colocalizing foci/cell**		**0.41**		

ROI = Region Of Interest
